# Hypertension at the nexus of veteran status, psychiatric disorders, and traumatic brain injury: Insights from the 2011 Behavioral Risk Factor Surveillance System

**DOI:** 10.1371/journal.pone.0298366

**Published:** 2024-03-18

**Authors:** Jacob P. DeBlois, Andrew S. London, Kevin S. Heffernan

**Affiliations:** 1 Department of Exercise Science, Syracuse University, Syracuse, NY, United States of America; 2 Department of Sociology, Syracuse University, Syracuse, NY, United States of America; King’s College London, UNITED KINGDOM

## Abstract

Variable military service-related experiences, such as combat exposure, psychiatric disorders (PD), and traumatic brain injuries (TBI), may differentially affect the likelihood of having health care professional-identified high blood pressure (i.e., hypertension). PURPOSE: Compare the odds of self-reported hypertension among non-combat and combat veterans with and without PD/TBI to non-veterans and each other. METHODS: We used data from men from the 2011 Behavioral Risk Factor Surveillance System and distinguished: non-veterans (*n* = 21,076); non-combat veterans with no PD/TBI (*n* = 3,150); combat veterans with no PD/TBI (*n* = 1,979); and veterans (combat and non-combat) with PD and/or TBI (*n* = 805). Multivariable, hierarchical logistic regression models included exogenous demographic, socioeconomic attainment and family structure, health behavior and conditions, and methodological control variables. RESULTS: One-third of men reported having been told at least once by a medical professional that they had high blood pressure. Bivariate analyses indicated that each veteran group had a higher prevalence of self-reported hypertension than non-veterans (design-based F = 45.2, *p*<0.001). In the fully adjusted model, no statistically significant differences in the odds of self-reported hypertension were observed between non-veterans and: non-combat veterans without PD/TBI (odds ratio [OR] = 0.92); combat veterans without PD/TBI (OR = 0.87); veterans with PD and/or TBI (OR = 1.35). However, veterans with PD and/or TBI had greater odds of reporting hypertension than both combat and non-combat veterans without PD/TBI (*p*<0.05). DISCUSSION: Military service-related experiences were differentially associated with a survey-based measure of hypertension. Specifically, veterans self-reporting PD and/or TBI had significantly higher odds of self-reporting hypertension (i.e., medical provider-identified high blood pressure).

## Introduction

High blood pressure, also known as hypertension, is a major public health concern. Nearly half (47.3%) of U.S. adults ≥20 years of age have hypertension, but many are unaware that they have it [1]. Individuals with hypertension are more likely to develop renal disease, cerebrovascular disease/cognitive decline, and heart failure, and to die prematurely [2]. Hypertension increases substantially with age and is higher among racial and ethnic minorities, persons with lower education and income, and persons who are obese, smoke, drink alcohol, and have low levels of physical activity [3–9]. Additionally, the American Heart Association recently released a scientific statement linking mental health conditions with increased risk for hypertension [10]. Identifying subgroups within the population with elevated risk for hypertension is important for the purposes of targeting prevention and increasing access to needed health care [11].

Veterans are a large and policy relevant subpopulation [12,13]. The U.S. Department of Veterans Affairs estimates that there were 19.5 million veterans living in the United States in 2020 [14]. Research examining the influence of military service on hypertension is conflicting, with some studies suggesting that prior military service is associated with increased risk for hypertension [15–17], while others report no veteran status difference in hypertension [18,19].

The military, as a total institution, has been conceptualized as both a protective and a risk-conferring environment for health across the life course [20]. As such, there are multiple mechanisms that might affect associations between military service experiences and hypertension. One mechanism that might be associated with lower risk of hypertension is the positive selection of the most physically and mentally fit at the time of induction or assignment to combat roles—the so-called “healthy soldier” and “healthy deployer” effects [21,22]. Some evidence suggests that the “healthy soldier” effect wanes over time and that veterans have similar, if not increased, morbidity and mortality rates relative to non-veterans later in the life course [23,24]. Consistent with a possible waning of the “healthy soldier” effect, one prospective longitudinal study showed that male veterans had better self-rated health than male non-veterans around retirement age, but their self-rated health declined more rapidly with increasing age [25].

Other mechanisms are more directly rooted in active-duty military service experiences, but may evolve and affect other hypertension-relevant processes and outcomes over the veteran life course [26]. Military personnel may experience a “military capital” effect [20] through: direct participation in a range of health-promoting activities during the active-duty period [27]; a reduction in certain types of substance use [28]; the development of social networks and occupational skills that can translate into better post-military jobs [29,30]; and access to post-service educational, health, and housing benefits available only to military veterans [31]. Service-related opportunities for career advancement are particularly important for youth from disadvantaged backgrounds because military service can produce positive turning points in their life-course trajectories by “knifing off” the influence of early-life disadvantages and providing a “bridging environment” to labor-market opportunities [32–35]. Thus, military service may lead to better socioeconomic status attainment across the life course via multiple mechanisms: 1) the influence of background characteristics related to selection into military service and socioeconomic attainment; 2) military service-related alterations of characteristics associated with later attainment; 3) network connections; 4) access to educational benefits through the G.I. Bill; and 5) the signaling effect of veteran status that influences hiring decisions, attainment, and income [26,36].

It is well-established that socioeconomic status/attainment can influence cardiovascular health and hypertension risk [5,37,38]. As a result of “military capital” that accrues during the active-duty period and through institutionalized benefits for veterans, some veterans may experience better family, health behavior, and health outcomes, as well as socioeconomic status, than non-veterans and veterans with different experiences in the military. These benefits of military service may allow some veterans to have lower odds of hypertension than non-veterans and veterans with different military service experiences.

However, there is also the potential for service-connected harm to reduce the potential benefits of “military capital” and/or directly contribute to variation in the risk for hypertension. Some aspects of military service are dangerous and contribute to long-lasting physical and psychological harm (i.e., the “military hazard” effect) [20]. Such service-connected influences on the risk of hypertension might result from: training injuries; accidents; deployment to conflict zones; in-theater exposure to radiation, chemicals, environmental toxicants (e.g., Agent Orange), and smoke; direct experience of combat; and military sexual trauma [39–43]. Other risks are less direct. For example, patterns of eating established during the early-adulthood, active-duty period, which involve high caloric intake in preparation for/response to high energy expenditure, may contribute to obesity among veterans who reduce their post-service physical activity levels without altering their eating habits [44,45]. Tobacco, alcohol, and drug use initiated or enhanced during the active-duty period may endure and influence later-life health risks [28,46–48]. Variable military service experiences have also been shown to differentially affect inadequate sleep and everyday drinking [49,50]. Recent evidence of a causal and potentially generalizable effect of military service on smoking behavior across the life course is noteworthy given the effect of smoking on a range of health outcomes, including hypertension [51]. This body of evidence suggests that health behaviors rooted in the active-duty period of the life course may contribute to the genesis of hypertension following separation from the military.

A large literature documents the negative consequences of some military service experiences on veterans’ mental health. Post-traumatic stress disorder (PTSD), anxiety, and depression are relatively common among military service members and veterans [52–56]. The etiology of these mental health conditions among veterans is complex and multifaceted, but it is often linked to traumatic experiences during combat. Such experiences may include bodily harm, as in the case of traumatic brain injuries (TBI), which have doubled from 2000 to 2011 [57]. Many psychiatric disorders (PD) and neuroanatomical disturbances overlap with TBI [58], and TBI can lead to the development of PD [59]. Therefore, differences in combat exposure, PD, and TBI might differentially affect the risk of hypertension among veterans [15,17,60,61]. Furthermore, potential neuroanatomical disruptions from PD/TBI may contribute to reduced socioeconomic attainment, thus further augmenting hypertension risk [62–65]. These findings highlight the potentially large influence of combat exposure, PD, and TBI on hypertension risk.

While combat exposure, PD, and TBI might differentially affect the risk of hypertension among veterans [15,17,60,61], it is worth noting that there is evidence of substantial physiological and/or psychological resilience and post-traumatic growth among some veterans [41,66,67]. Low levels of resilience (i.e., the reduced ability to minimize cardiovascular allostatic load relative to those with higher levels of resilience [68]), has been associated with hypertension via vascular structure and functional changes [69,70], and through the adoption of health behaviors that increase hypertension risk [71]. In contrast, veterans with a high degree of resilience and post-traumatic growth may adopt better health behaviors, and experience better health and well-being across the life course.

Veterans are not a monolithic group. As hypertension is one of the leading contributors to death in the United States among non-veteran civilians and veterans alike [1], it is important to better understand variation in the risk of hypertension at the nexus of veteran, combat, and PD/TBI statuses. Therefore, the purpose of this study was to compare the odds of self-reporting hypertension among veterans with and without combat experience and with and without PD/TBI relative to each other and non-veterans. Consistent with prior research [49,50], we hypothesized that heterogeneous military service-related experiences would be differentially associated with hypertension and that veterans with PD/TBI would have a significantly higher likelihood of hypertension than non-veterans and both non-combat and combat veterans without PD/TBI.

## Materials and methods

### Study design

We used public-use data from the Centers for Disease Control and Prevention’s (CDC) 2011 Behavioral Risk Factor Surveillance System (BRFSS) (https://www.cdc.gov/brfss/index.html; accessed 27 July 2022) [72]. The public-use dataset is de-identified. The full BRFSS is an annual, population-representative survey of all 50 states, Washington, D.C., Guam, the U.S. Virgin Islands, and Puerto Rico, with each location fielding a standard core survey that can be supplemented with one or more optional topic modules. The BRFSS utilizes geographic stratification and a multi-stage sampling design within states/territories for data collection. We used data from 2011 because it was the only BRFSS that included a core question that asks participants if they have ever been told by a health care professional that they have high blood pressure and the optional Veteran Health module, which included separate measures of combat exposure, PD, and TBI. The analytic sample included participants from Alaska, Kansas, Louisiana, Maine, Nebraska, Nevada, New Jersey, North Carolina, and Tennessee, which were the states that elected to field the Veteran Health module in 2011. Very few women reported combat exposure. Therefore, we only included men in the analytic sample.

The BRFSS surveys were conducted by the CDC with full ethical approval and an informed consent process. Syracuse University’s Office of Research Integrity and Protections waives the requirement for Institutional Review Board review for de-identified, public use data that does not require a data use agreement; as such, informed consent and further ethical review was not required.

### Measures

#### Hypertension

In 2011, the core survey included a question about high blood pressure/hypertension: “*Have you EVER been told by a doctor*, *nurse or other health professional that you have high blood pressure*?” From responses to this question, the CDC derived a dichotomous variable and made it available in the public-use BRFSS dataset. Participants who answered in the affirmative (“Yes”) were coded as 1, whereas those who answered in the negative (“No”) were coded as 0. Individuals who responded that they were “*told [their blood pressure was] borderline high or pre-hypertensive*” were included in the zero (no high blood pressure/hypertension) category (unweighted *n* = 459 in the analytic sample). We used this CDC-derived variable as the dependent variable in the analyses of hypertension that follow.

#### Military service experiences–veteran status, combat exposure, PD, and TBI

Data regarding military service-related experiences were obtained from the core survey as well as the Veteran Health module. The core survey included the question: “*Have you ever served on active duty in the United States Armed Forces*, *either in the regular military or in a National Guard or military reserve unit*? *Active duty does not include training for the Reserves or National Guard*, *but DOES include activation*, *for example*, *for the Persian Gulf War*.” Participants in states that included the Veteran Health module who answered “Yes” were asked a series of military service-related questions that included distinct questions about combat exposure, PD, and TBI: “*Did you ever serve in a combat or war zone*?”; “*Has a doctor or other health professional ever told you that you have depression*, *anxiety*, *or post-traumatic stress disorder (PTSD)*?”; “*A traumatic brain injury may result from a violent blow to the head or when an object pierces the skull and enters the brain tissue*. *Has a doctor or other health professional ever told you that you have suffered a traumatic brain injury (TBI)*?” From these survey questions, we derived a four-group independent variable that measures military service experiences: non-veterans; non-combat veterans with no PD or TBI; combat veterans with no PD or TBI; and veterans (non-combat and combat) with a PD and/or TBI. Veterans with only PD, only TBI, as well as participants with both PD and TBI were included in this fourth category. We combined non-combat and combat veterans in this final group due to the relatively small number of non-combat veterans with a PD and/or TBI diagnosis (*n* = 325 with an additional *n* = 4 missing combat status with PD and/or TBI in the analytic sample).

#### Covariates

We included four sets of covariates in the analysis: exogenous demographic variables; socioeconomic attainment and family structure variables; health behavior and conditions variables; and methodological control variables (see Table 1 for details; specific information about the survey questions is available at https://www.cdc.gov/brfss/questionnaires/pdf-ques/2011brfss.pdf). The exogenous demographic variables included the participant’s age, race, and Hispanic ethnicity; we considered these distinctly because they are exogenous to both military service and hypertension. The socioeconomic attainment and family structure variables may be associated with exogenous factors that select people into military service, but can also be affected by military service, combat exposure, PD, and TBI [26,73]. These covariates included: education; employment status; household income; marital status; and number of children in the household. Health behaviors and conditions that are proximal to and often comorbid with hypertension, and may be associated with military service, combat exposure, PD, and TBI, were included as a separate set of covariates. These included: current smoking status; heavy alcohol consumption; exercise in the past 30 days; body mass index (BMI); ever been told they have diabetes by a health care professional; and ever been told they had a heart attack by a health care professional. The final set of covariates included methodological control variables. We included state of residence to account for variation in state-specific policies and conditions, and survey year to account for the fact that some interviews were conducted in 2012 even though the sample was drawn in 2011.

**Table 1:** Population description, men in selected states, 2011 Behavioral Risk Factor Surveillance System.

	Total		Non-Veteran (%)		Non-Combat Veteran, No PD/TBI (%)	Combat Veteran, No PD/TBI (%)	Veteran with a PD and/or TBI (%)
	Weighted (%)	Unweighted (*N*)					
Sample size *N (*%)	100	27,010	21,076 (82.3)		3,150 (8.8)	1,979 (6.0)	805 (2.9)
**EXOGENOUS DEMOGRAPHICS**							
**Age (yrs)†**							
18–29	21.4	1,574	24.5		4.6	8.1	13.7
30–39	19.1	2,782	20.6		10.7	12.7	17.6
40–49	19.7	4,641	21.2		12.8	13.4	11.5
50–59	18.4	6,824	19.1		17.1	11.1	17.5
60–69	12.6	6,086	9.8		22.0	28.5	29.5
70+	8.8	5,098	4.9		32.8	26.3	10.2
**Race†**							
White	74.2	23,553	73.0		81.8	79.0	76.7
Black	14,6	1,659	14.7		13.7	15.1	12.5
Asian/Pacific Islander	3.1	383	3.6		0.2	1.3	0.9
Native American/Alaskan	1.6	413	1.5		0.9	1.3	4.4
Other	4.8	628	5.4		1.2	2.0	3.7
Multiracial	1.8	374	1.8		2.3	1.2	1.8
**Hispanic Identity†**							
Non-Hispanic	91.8	26,002	90.8		95.8	97.2	93.8
Hispanic	8.2	1,008	9.1		4.2	2.8	6.2
**SOCIOECONOMIC AND FAMILY STRUCTURE**							
**Education†**							
Less than High School	14.7	2,193	15.9		9.8	8.0	33.2
High School	30.6	8,092	30.7		32.4	26.2	13.0
Some College	27.8	6,603	25.7		36.1	36.8	29.6
College or More	27.0	10,122	27.8		21.6	29.0	24.2
**Employment Status†**							
Employed	61.0	15,757	64.4		45.5	50.6	33.2
Unemployed	10.8	1,600	11.6		6.8	4.4	13.0
Not in Labor Force	20.7	7,881	16.4		43.3	42.1	29.6
Disabled, Unable to Work	7.6	1,772	7.7		4.4	2.8	24.2
**Household Income†**							
$1 - $25,000	23.4	5,547	23.7		20.8	17.8	35.2
$25,001 - $75,000	34.5	10,677	32.6		43.8	43.2	42.0
≥$75,001	28.2	8,337	29.3		23.3	26.2	13.7
Missing	13.9	2,449	14.4		12.2	12.8	9.1
**Marital Status†**							
Married	55.0	17,589	51.8		71.1	72.3	61.8
Partnered	3.6	483	4.2		0.4	1.4	1.4
Divorced/Separated	10.5	3,646	10.0		10.4	11.2	22.4
Widowed	3.2	1,738	2.1		8.9	8.7	3.9
Never Married	27.8	3,554	32.0		9.2	6.4	10.6
**Children in Household†**							
No Children	61.1	19.684	58.7		74.5	70.7	67.6
1 Child	16.4	2,844	17.5		9.6	9.7	17.8
2 Children	14.5	2,818	15.5		9.1	12.2	8.7
≥3 Children	8.0	1,664	8.3		6.8	7.4	5.9
**HEALTH BEHAVIORS AND CONDITIONS**							
**Current Smoking Status†**							
Never Smoked	49.6	12,913	52.2		38.8	36.1	35.4
Previous Smoker	27.1	9,462	23.7		42.4	45.8	38.6
Smokes Some Days	6.2	1,102	6.4		5.2	5.5	4.5
Smokes Every Day	17.2	3,533	17.7		13.7	12.6	21.5
**Heavy Drinker**							
No	93.6	25,393	93.4		95.2	94.3	92.2
Yes	6.4	1,617	6.6		4.8	5.7	7.8
Exercised in Past 30 Days*							
No	26.3	7,199	25.9		31.4	22.4	31.1
Yes	73.7	19,811	74.2		68.6	77.6	68.9
**Body Mass Index (BMI)** **†**							
Underweight	1.2	176	1.3		0.3	0.8	1.1
Normal Weight	28.3	6,468	29.2		22.9	26.9	22.1
Overweight	41.6	12,119	40.8		47.4	46.7	36.5
Obese	28.9	8,247	28.7		29.4	25.6	40.2
**Ever Told have Diabetes†**							
No	89.9	23,321	91.3		83.3	85.1	81.4
Yes	10.1	3,689	8.7		16.7	14.9	18.7
**Ever Told had a Heart Attack†**							
No	94.2	24,732	95.4		89.1	89.9	84.3
Yes	5.9	2,278	4.6		11.0	10.1	15.7
**METHODLOGICAL CONTROLS**							
**State of Residence†**							
Alaska	2.0	1,249	1.8		2.6	3.1	2.3
Kansas	7.5	4,943	8.0		4.8	5.4	4.6
Louisiana	12.4	2,719	11.9		15.8	13.9	13.2
Maine	3.3	3,181	3.6		2.0	1.4	2.9
Nebraska	4.4	5,198	5.0		1.5	1.5	1.4
Nevada	7.0	1,387	6.4		9.4	11.0	8.5
New Jersey	20.3	3,563	23.3		7.2	5.4	4.9
North Carolina	26.2	3,274	24.2		33.5	37.7	36.4
Tennessee	17.0	1,496	15.8		23.1	20.7	25.9
**Data Collection Year**							
2011	98.1	26,697	98.3		97.4	97.1	98.1
2012	1.9	313	1.7		2.6	2.9	1.9
**TOTAL SAMPLE**	100	27,010					

*Significant at *p*<0.01

†Significant at *p*<0.001.

### Statistical analyses

The analytic sample includes 27,010 participants with complete data on all variables. We examined the characteristics of the population represented by the weighted sample and estimated the bivariate association between our four-group military service experience variable and ever reported hypertension. We assessed the statistical significance of bivariate associations using a design-based F statistic. Additionally, we estimated four multivariable, hierarchical logistic regression models predicting the odds of ever reporting hypertension: Model 1 included the military service experiences and methodological control variables; Model 2 added the exogenous demographic variables to Model 1; Model 3 added to Model 2 the potentially mediating socioeconomic attainment and family structure variables; and Model 4 added the potentially mediating health behaviors and conditions variables. Post-hoc Wald tests were used to assess differences between groups of veterans with different military service experiences.

Data were weighted for all analyses and the standard errors were adjusted to take the complex sample design into account. Due to the design of the BRFSS, geographic stratification and selection of primary sampling units produces clustering of elements at subsequent stages. Such sample design-based clustering violates the independence of observations assumption necessary for maximum likelihood estimation. Thus, design-based F and Wald statistics were used [74]. Significance was set at an alpha level of *p*<0.05. All analyses were performed using the SVY commands in Stata 14.1 (StataCorp; College Station, TX).

## Results

### Population description

Table 1 presents a description of the total population and the four military service experience subpopulations. Overall, 17.7% of the study population were veterans. Approximately 60% were aged 18 to 49 years. A majority (three-quarters) self-identified as White and were of non-Hispanic ethnicity. With respect to socioeconomic and family statuses, 45.3% reported high school education or less, 61.0% were employed, and 57.9% reported household income of $75,000 or less. Fifty-five percent were married and 61.1% had no child living in their household at the time of the survey. With respect to health behaviors and conditions, 17.2% were everyday smokers, while another 6.2% smoked some days. A small minority (6.4%) were classified as heavy alcohol consumers and approximately three-quarters had exercised in the past 30 days. The majority were overweight (41.6%) or obese (28.9%). Ten percent reported that they have diabetes and 5.9% reported that they had a heart attack. Four states—Louisiana, New Jersey, North Carolina, and Tennessee—accounted for about 76% of the population represented by the analytic sample.

As shown in Table 1, the veteran subpopulations had substantially higher concentrations of older adults than the non-veteran population, and also had higher concentrations of persons who self-identified as White and as non-Hispanic (*p*<0.001). Veteran subpopulations had higher concentrations of persons with some college than the non-veteran population (*p*<0.001). Perhaps reflecting their older age, on average, the veteran subpopulations had lower concentrations of employed persons than the non-veteran population (*p*<0.001). Notably, veterans with a PD and/or TBI had a substantially higher concentration of persons reporting that they were disabled and unable to work (24.2%) than any of the other groups (*p*<0.001). Compared to the non-veteran population, the veteran subpopulations had higher concentrations of persons reporting household income in the $25,000-$75,000 range (*p*<0.001). The veteran subpopulations also reported higher concentrations of currently married persons and higher concentrations of persons with no children in their households (perhaps a reflection of their older age; *p*<0.001).

Health behaviors and conditions varied across the military service experience subpopulations, with some heterogeneity evident among veterans with different experiences. The veteran subpopulations had substantially lower concentrations of persons who had never smoked, although they had higher concentrations of previous smokers (*p*<0.001). Compared to non-veterans, non-combat veterans with no PD/TBI and veterans with a PD and/or TBI had higher concentrations of persons who reported no exercise in the previous 30 days (*p*<0.01). In contrast, combat veterans with no PD/TBI had a lower concentration of persons who reported no exercise in the past 30 days compared with non-veterans (*p*<0.001). With respect to BMI, the four groups had similar levels of overweight and obesity. However, compared with non-veterans, the concentrations of overweight persons were higher among non-combat veterans and combat veterans with no PD/TBI (*p*<0.001). Additionally, the concentrations of obesity were higher among non-combat veterans with no PD/TBI and veterans with a PD and/or TBI relative to non-veterans (*p*<0.001). Again, possibly reflecting their older age, on average, the veteran subpopulations had much higher concentrations of persons with diabetes and who have had a heart attack than the non-veteran population (*p*<0.001).

### Hypertension

**Fig 1:**
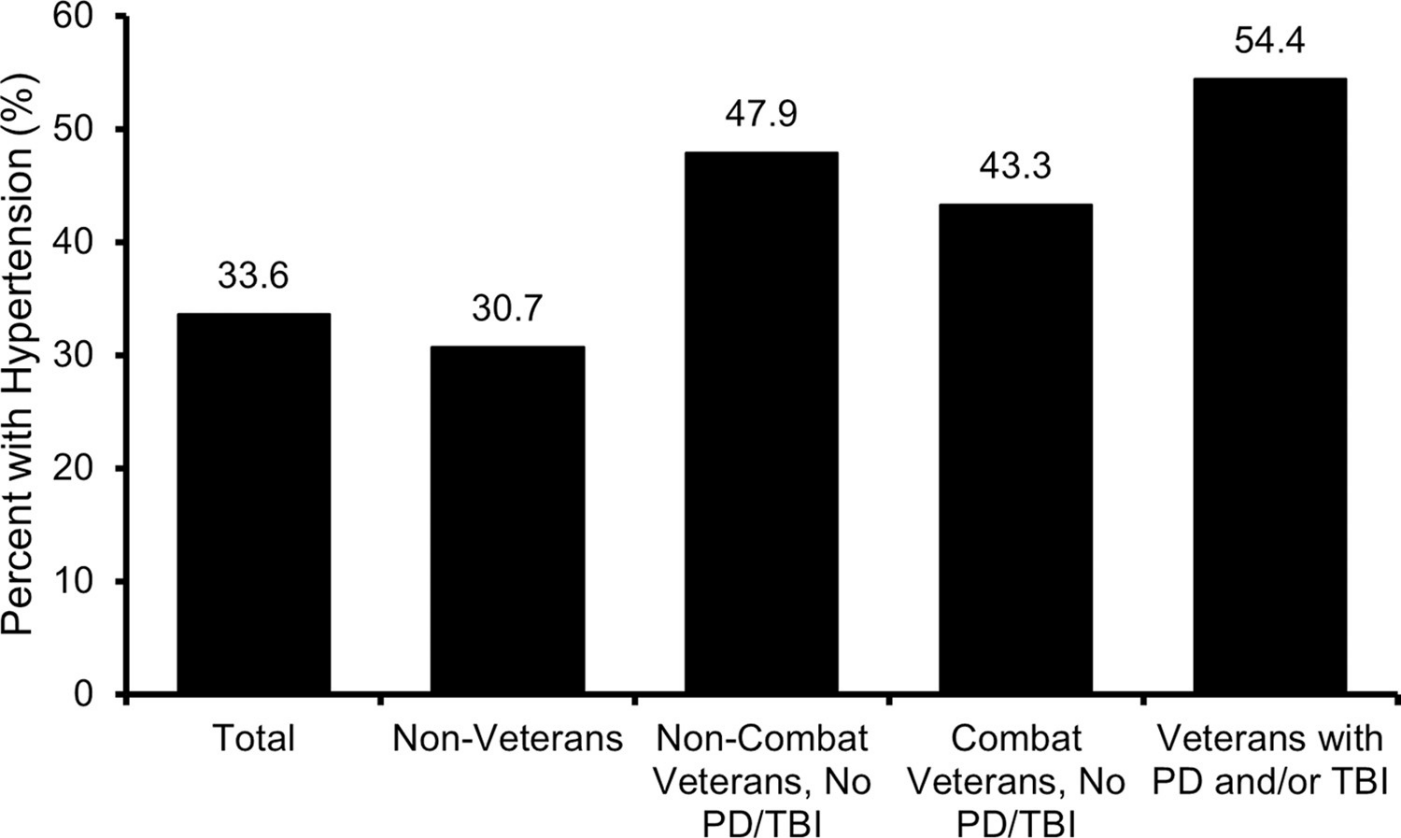
Percent of men with hypertension, overall and by military service experience status (N = 27,010). Self-reported hypertension was more prevalent among veterans than non-veterans (design-based F = 45.2, p<0.001).

Fig 1 presents prevalence of self-reported hypertension across the military service experience groups. Overall, 33.6% reported hypertension, but hypertension was more prevalent among veterans than non-veterans (design-based F = 45.2, *p*<0.001). We conducted multivariable analyses to determine if these differences in the likelihood of hypertension were in part due to differences in the composition of the military service experience subpopulations.

### Multivariable analyses

Table 2 presents results from a series of four nested multivariable logistic regression analyses of men’s likelihood of hypertension. The results from Model 1, which just includes the four-category military service experiences and methodological control variables, were consistent with the bivariate results shown in Fig 1 in that each of the veteran groups had significantly higher odds of ever reporting hypertension relative to non-veterans (*p*<0.001). Specifically, the odds of hypertension were 98% higher among non-combat veterans with no PD/TBI, 66% higher among combat veterans with no PD/TBI, and 2.56 times higher among veterans with a PD and/or TBI relative to non-veterans. Post-hoc testing indicated that veterans with a PD and/or TBI had significantly greater odds of hypertension than combat veterans with no PD/TBI (*p*<0.05).

**Table 2:** Multivariate logistic regression analysis of hypertension among men.

	Model 1		Model 2		Model 3		Model 4	
Variable (Reference)	β (SE)	OR	β (SE)	OR	β (SE)	OR	β (SE)	OR
**Military Service Experiences** (Non-Veteran)								
Non-Combat Veteran, No PD/TBI	0.681 (0.083)	1.98^‡^	-.096^b^ (0.080)	0.91	-.076^b^ (0.080)	0.93	-0.087^b^ (0.084)	0.92
Combat Veteran, No PD/TBI	.509^c^ (0.097)	1.66^‡^	-.201^c^ (0.098)	0.82*	-.166^c^ (0.102)	0.85	-0.141^c^ (0.107)	0.87
Veteran with a PD and/or TBI	.939^c^ (0.166)	2.56^‡^	.618^b,c^ (0.175)	1.86^‡^	.405^b,c^ (0.170)	1.50*	0.298^b,c^ (0.181)	1.35
**EXOGENOUS DEMOGRAPHICS**								
**Age** (18–29 years)								
30–39 years	--	--	0.597 (0.156)	1.82^‡^	0.52 (0.167)	1.68^†^	0.308 (0.166)	1.36
40–49 years	--	--	1.373 (0.143)	3.95^‡^	1.201 (0.153)	3.32^‡^	0.921 (0.152)	2.51^‡^
50–59 years	--	--	1.987 (0.135)	7.29^‡^	1.662 (0.153)	5.27^‡^	1.358 (0.154)	3.89^‡^
60–69 years	--	--	2.494 (0.138)	12.11^‡^	2.084 (0.157)	8.04^‡^	1.714 (0.161)	5.55^‡^
70+ years	--	--	2.727 (0.141)	15.29^‡^	2.287 (0.164)	9.84^‡^	1.941 (0.172)	6.97^‡^
**Race** (White)								
Black	--	--	0.295 (0.095)	1.34^†^	0.258 (0.101)	1.29*	0.218 (0.104)	1.24*
Asian/Pacific Islander	--	--	-0.323 (0.204)	0.72	-0.207 (0.210)	0.81	-0.062 (0.220)	0.94
Native American/Alaskan	--	--	0.446 (0.288)	1.56	0.331 (0.285)	1.39	0.209 (0.318)	1.23
Other	--	--	-0.151 (0.199)	0.86	-0.11 (0.200)	0.90	-0.006 (0.204)	0.99
Multiracial	--	--	0.389 (0.238)	1.48	0.266 (0.220)	1.30	0.256 (0.216)	1.29
**Hispanic Identity** (Non-Hispanic)								
Hispanic	--	--	-0.205 (0.158)	0.81	-0.291 (0.161)	0.75	-0.401 (0.162)	0.67*
**SOCIOECONOMIC AND FAMILY STRUCTURE**								
**Education** (Less than High School)								
High School	--	--	--	--	0.02 (0.107)	1.02	0.020 (0.116)	1.02
Some College	--	--	--	--	0.079 (0.113)	1.08	0.082 (0.120)	1.09
College or More	--	--	--	--	-0.209 (0.113)	0.81	-0.070 (0.124)	0.93
**Employment Status** (Employed)								
Unemployed	--	--	--	--	0.160 (0.120)	1.17	0.121 (0.120)	1.13
Not in Labor Force	--	--	--	--	0.099 (0.085)	1.10	0.057 (0.090)	1.06
Disabled, Unable to Work	--	--	--	--	0.842 (0.129)	2.32^‡^	0.656 (0.138)	1.93^‡^
**Household Income** ($1 - $25,000)								
$25,001 - $75,000	--	--	--	--	0.000 (0.089)	1.00	-0.021 (0.092)	0.98
≥$75,001	--	--	--	--	-0.182 (0.103)	0.83	-0.189 (0.108)	0.83
Missing	--	--	--	--	-0.238 (0.116)	0.79*	-0.218 (0.122)	0.80
**Marital Status** (Married)								
Partnered	--	--	--	--	0.132 (0.219)	1.14	0.179 (0.216)	1.20
Divorced/Separated	--	--	--	--	0.005 (0.087)	1.01	0.069 (0.092)	1.07
Widowed	--	--	--	--	0.103 (0.107)	1.11	0.087 (0.112)	1.09
Never Married	--	--	--	--	-0.362 (0.106)	0.70^†^	-0.269 (0.108)	0.76*
**Children in Household** (No Children)								
1 Child	--	--	--	--	-0.283 (0.096)	0.75^†^	-0.279 (0.100)	0.76^†^
2 Children	--	--	--	--	-0.296 (0.109)	0.74^†^	-0.259 (0.113)	0.77*
≥3 Children	--	--	--	--	-0.430 (0.128)	0.65^†^	-0.428 (0.129)	0.65^†^
**HEALTH BEHAVIORS AND CONDITIONS**								
**Current Smoking Status** (Never Smoked)								
Previous Smoker	--	--	--	--	--	--	0.202 (0.072)	1.22^†^
Smokes Some Days	--	--	--	--	--	--	0.096 (0.147)	1.10
Smokes Every Day	--	--	--	--	--	--	0.085 (0.096)	1.09
**Heavy Drinker** (No)								
Yes	--	--	--	--	--	--	0.075 (0.111)	1.08
**Exercised in Past 30 Days** (No)								
Yes	--	--	--	--	--	--	-0.089 (0.072)	0.91
**Body Mass Index (BMI)** (Normal Weight)								
Underweight	--	--	--	--	--	--	-0.028 (0.459)	0.97^‡^
Overweight	--	--	--	--	--	--	0.529 (0.076)	1.70^‡^
Obese	--	--	--	--	--	--	1.158 (0.087)	3.18^‡^
**Ever Told have Diabetes** (No)								
Yes	--	--	--	--	--	--	0.960 (0.090)	2.61^‡^
**Ever Told had a Heart Attack** (No)								
Yes	--	--	--	--	--	--	0.784 (0.130)	2.19^‡^
**METHODLOGICAL CONTROLS**								
**State of Residence** (Alaska)								
Kansas	0.010 (0.099)	1.01	-0.061 (0.118)	0.94	-0.106 (0.117)	0.90	-0.18 (0.122)	0.84
Louisiana	0.356 (0.110)	1.43^†^	0.297 (0.13)	1.36*	0.225 (0.129)	1.25	0.129 (0.135)	1.14
Maine	0.151 (0.103)	1.16	-0.003 (0.123)	1.00	-0.100 (0.122)	0.91	-0.135 (0.127)	0.87
Nebraska	-0.118 (0.103)	0.89	-0.197 (0.122)	0.82	-0.231 (0.121)	0.79	-0.278 (0.126)	0.76*
Nevada	0.103 (0.137)	1.11	0.134 (0.161)	1.14	0.080 (0.161)	1.08	0.065 (0.164)	1.07
New Jersey	-0.026 (0.104)	0.97	-0.124 (0.125)	0.88	-0.097 (0.122)	0.91	-0.125 (0.128)	0.88
North Carolina	0.086 (0.109)	1.09	-0.004 (0.129)	1.00	-0.072 (0.128)	0.93	-0.083 (0.133)	0.92
Tennessee	0.341 (0.123)	1.41^†^	0.256 (0.139)	1.29	0.147 (0.136)	1.16	0.091 (0.141)	1.10
**Data Collection Year** (2011)								
2012	-0.279 (0.220)	0.76	-0.289 (0.195)	0.75	-0.299 (0.192)	0.74	-0.380 (0.206)	0.68
**Constant**	-0.934 (0.09)	0.39^‡^	-2.203 (0.163)	0.11^‡^	-1.724 (0.219)	0.18^‡^	-2.226 (0.237)	0.11^‡^

Significance Levels

* = p < 0.05

† = p < 0.01

‡ = p < 0.001.

^**a**^Significant difference between non-combat veterans with no PD or TBI and combat veterans with no PD or TBI (p < 0.05).

^**b**^Significant difference between non-combat veterans with no PD or TBI and veterans with a PD and/or TBI (p < 0.05).

^**c**^Significant difference between combat veterans with no PD or TBI and veterans with a PD and/or TBI (p < 0.05).

Model 2 added the exogenous demographic control variables and indicated the odds of ever reporting hypertension were not significantly different between non-combat veterans with no PD/TBI and non-veterans. However, relative to non-veterans, the odds of hypertension were significantly lower among combat veterans with no PD/TBI (OR = 0.82, *p*<0.05) and significantly higher among veterans with a PD and/or TBI (OR = 1.86, *p*<0.001). Moreover, post-hoc tests indicated that the odds of hypertension were significantly higher among veterans with a PD and/or TBI than both non-combat and combat veterans with no PD/TBI (*p*<0.05).

Model 3 added to Model 2 the potentially mediating socioeconomic attainment and family status variables. The results from Model 3 mostly aligned with the results from Model 2: non-combat veterans with no PD/TBI were not significantly different from non-veterans, while veterans with a PD and/or TBI maintained statistically significantly higher odds of hypertension compared with non-veterans (OR = 1.50, *p*<0.05). However, the odds of hypertension were similar between non-veterans and combat veterans with no PD/TBI. Veterans with a PD and/or TBI had higher odds of hypertension compared to both of the other veteran groups (*p*<0.05).

The fully adjusted Model 4 included the potentially mediating health behaviors and conditions variables along with all other covariates. Adding these variables resulted in similar odds of hypertension between veterans with a PD and/or TBI and non-veterans (*p*>0.05). However, post-hoc tests indicated that the odds of hypertension among veterans with a PD and/or TBI were significantly higher than the odds among non-combat and combat veterans with no PD/TBI (*p*<0.05). Thus, none of the veteran subpopulations differed from non-veterans once we included all the potentially mediating variables in the model, but veterans with a PD and/or TBI were more likely than non-combat and combat veterans with no PD or TBI to report hypertension.

## Discussion/Conclusions

The purpose of this study was to compare the odds of ever self-reporting hypertension among groups of veterans with different military experiences (i.e., combat experience, PD/TBI) to each other and to non-veterans. We hypothesized that military service-related experiences would be differentially associated with a survey-based measure of hypertension (i.e., ever told by a health care professional that you have high blood pressure) and that veterans with self-reported PD/TBI would have significantly higher odds of self-reported hypertension than veterans without PD/TBI and non-veterans. Results partially support these hypotheses.

Results from a model that includes the military service experience variable and exogenous demographic and methodological controls (Model 2) provide evidence of heterogenous and countervailing influences of military service experiences on hypertension. Combat veterans with no PD/TBI had significantly lower odds of reporting hypertension than non-veterans, while veterans with PD and/or TBI had significantly higher odds of reporting hypertension than non-veterans, non-combat veterans with no PD/TBI, and combat veterans with no PD/TBI. When potentially mediating socioeconomic attainment, family status, health behaviors, and health conditions variables were added to the model (Model 4), none of the three veteran subpopulations differed from non-veterans, but heterogeneity in the consequences of military service experiences for hypertension remained evident. Specifically, veterans with PD and/or TBI had greater odds of hypertension than both combat and non-combat veterans without PD/TBI. Notably, there was no difference in the odds of hypertension between non-combat veterans with no PD/TBI and combat veterans with no PD/TBI.

Taken together, these results suggest that there are heterogenous and countervailing consequences of military service experiences for hypertension. Neither veteran status nor combat status *per se* are associated with higher odds of hypertension; however, veterans who have a diagnosis of PD and/or TBI are more likely than both non-combat and combat veterans with no PD/TBI to report having hypertension. Stated otherwise, non-combat and combat veterans with no PD/TBI are significantly less likely than veterans (non-combat and combat combined) with PD and/or TBI to report hypertension. Thus, what seems to matter for veterans and hypertension is PD/TBI.

Negative psychological health (e.g., anxiety, depression, PTSD) has been linked to behavioral changes and biological processes that may be detrimental for cardiovascular health, thus increasing the risk for cardiovascular diseases and hypertension [10]. Specifically, individuals with PD may have a more robust cardiovascular reactivity to stressful situations [75,76] leading to autonomic dysregulation [77], arterial calcification [78], and endothelial dysfunction [79,80]. Meanwhile, TBI, depending on the cerebral location, if the injury was localized to one cerebral location or diffuse across several, and injury severity, can result in axonal injury [81], neurovascular detriments [82], electrophysiological changes [83], and an uncoupling between the cardiovascular and autonomic nervous system [84]. The neuroanatomical disruptions from TBI have been linked to the development of PD [59], anduncoupling of autonomic control over the cardiovascular system can lead to reduced baroreflex sensitivity [85], thereby linking TBI to hypertension. Due to the prevalence of PD among veterans [52–55,86] and the growth in the number of veterans who have experienced TBI in recent years [57], the presence of PD and/or TBI among veterans appears to be an important factor for hypertension among these individuals. PD/TBI might also matter for hypertension among non-veterans; however, a limitation of this study is that we are unable to measure PD/TBI among non-veterans in the BRFSS. It will be important for future comparative research to measure PD/TBI consistently in both veteran and non-veteran populations.

Our fully adjusted model showed that military combat did not influence the odds of hypertension among men who served in the military. While this finding is substantiated by others [18], this is an issue of active debate in the literature. The root of discrepant findings may stem from how questions about military combat are asked. A dichotomous assessment of combat experience (“combat” vs. “non-combat”) may not be enough to fully measure the influence of combat on hypertension and other health outcomes. For example, Granado et al. found 33% greater odds of developing hypertension with *multiple* combat deployments relative to non-combat deployed service members, whereas those with a single combat deployment did not differ in incident hypertension risk (adjusted OR: 1.02, 95% confidence interval [CI]: 0.69–1.51) [17]. Additionally, those who witnessed death regardless of the number of combat deployments were 43–50% more likely to develop hypertension [17]. Combat-related injuries can further contribute to hypertension risk. Data from the Millennium Cohort showed that service members with combat deployment *and injury* had an adjusted OR of 1.46 (95% CI: 1.07–2.00) for the development of hypertension relative to service members without combat and without injury [15]. It may be that the type and severity of combat-related stress/injury plays a larger role in hypertension development than being deployed to a combat zone itself. The BRFSS data set does not allow for the same type of combat experience assessment, which limits our ability to fully determine if military combat contributes to hypertension risk.

There are several limitations to this study that may influence interpretation of our findings. First, hypertension was self-reported. Although it is common for researchers to use self-report data when studying hypertension [87–89], hypertension status may be underestimated by self-report compared with hypertension status determined from measurement of brachial blood pressure [90–94]. It is interesting to note that the estimated prevalence of hypertension in the population represented by our analytic sample (33.6%) is consistent with estimates obtained from NHANES 2011–2012 (29.7%), which used measured blood pressure [95]. This similarity provides some evidence to support the external validity of our analyses even though the analytic sample we used largely comes from four specific states. Nonetheless, future research should examine the issues addressed in this paper using measured blood pressure and nationally representative samples.

Second, the National Center for Veterans Analysis and Statistics reports that, in 2011, men who were veterans had a greater prevalence of private and public health insurance coverage, including Medicare, and a lower uninsured rate than non-veteran men [96]. Therefore, a larger share of the non-veteran population may be unaware of their hypertension status and may have reported themselves to not have high blood pressure (i.e., hypertension). It is also possible that recall and social desirability biases may have contributed to an under-reporting of hypertension [97].

Third, the BRFSS is cross-sectional and therefore does not allow for analyses focused on the development of hypertension as people age. Additionally, it is not possible to assess the dynamic aspects of factors that influence hypertension risk over time. For example, the BRFSS asks about current socioeconomic status, but does not take into account changes in socioeconomic status over time. Future studies that examine the issues addressed in this study using longitudinal data will be important to more fully elucidate the influence of military service experiences on hypertension.

Fourth, the BRFSS questionnaire and Veteran Health module do not include specific details regarding combat experiences, TBI, or PD. Therefore, it is difficult to ascertain exactly what combat exposures were experienced by the veterans and how those experiences influenced hypertension risk. Severity, cerebral location, localized or diffuse TBI, and timing of TBI was also not measured. Thus, we are unable to determine if hypertension is associated with these aspects of TBI in the veteran population. Similarly, participants were asked with one question if they were ever told by a health care professional that they had “anxiety, depression, or post-traumatic stress disorder (PTSD).” For this reason, we are unable to determine which psychological conditions are related to hypertension among veterans. Importantly, for future research, the non-veteran group was not asked about the presence of PD/TBI. Thus, it is unclear how many participants in this group had a PD and/or TBI and if that influenced hypertension differently from those without PD/TBI.

Finally, due to weakened statistical power for sex-by-group comparisons, women were not included in this analysis. Future work should examine how military experiences specifically influence the cardiovascular and hypertensive health of women veterans.

In conclusion, this study found that military combat and PD/TBI do not increase the odds of self-reported hypertension prevalence in veteran men relative to non-veteran men *per se*. However, veterans with PD and/or TBI do have increased odds of hypertension relative to veterans without PD/TBI regardless of their combat experience. These findings suggest that mental and cerebrovascular health may play a large role in hypertension among veteran men. Future work should examine which PD (e.g., anxiety, depression, and/or PTSD) most influences hypertension among veteran men, if similar patterns of hypertension based on military experiences exist among veteran women, and interventions for TBI treatment to delay/halt the development of hypertension.
